# 

**Published:** 1883-01

**Authors:** 


					﻿Vol. Ill
JANUARY, 1883/
No. 1.
THE
HOMEOPATHIC PHYSICIAN,
A MONTHLY JOURNAL OF MEDICAL SCIENCE.
“ Magna est vefttax,,el proevalebtlJ' '
HJ. CT. LEE, LZE. ID.,
EDITOR.
CONTENTS:
(See inside of Cover.)
PHILADELPHIA:
No. 2109 CHESTNUT STREET.
□STBJ-W YOmt:
A. I* CHATTERTON PUBLISHING COMPANY, Publisher’s Agents.
r. o asr r> o jst ••
ALFRED HEATH & CO., Sole Agents for Great Britain,
114 Ebury Street, Eaton Square.
Yearly Subscription, $2.50. invariably in advance.
Entered at the Philadelphia Post-Office as Second Class Mail Matter.
				

